# Frontal theta brain activity varies as a function of surgical experience and task error

**DOI:** 10.1136/bmjsit-2020-000040

**Published:** 2020-11-09

**Authors:** Ahmed Mohammed Balkhoyor, Muhammad Awais, Shekhar Biyani, Alexandre Schaefer, Matt Craddock, Olivia Jones, Michael Manogue, Mark A Mon-Williams, Faisal Mushtaq

**Affiliations:** 1 School of Dentistry, University of Leeds, Leeds, UK; 2 Faculty of Dentistry, Umm Al-Qura University, Makkah, Saudi Arabia; 3 School of Psychology, University of Leeds, Leeds, UK; 4 Urology, St James University Hospital, Leeds, UK; 5 Department of Psychology, Jeffrey Cheah School of Medicine and Health Sciences, Monash University, Selangor, Malaysia; 6 School of Psychology, Lincoln University, Lincoln, UK

**Keywords:** dental devices, learning curve

## Abstract

**Objective:**

Investigations into surgical expertise have almost exclusively focused on overt behavioral characteristics with little consideration of the underlying neural processes. Recent advances in neuroimaging technologies, for example, wireless, wearable scalp-recorded electroencephalography (EEG), allow an insight into the neural processes governing performance. We used scalp-recorded EEG to examine whether surgical expertise and task performance could be differentiated according to an oscillatory brain activity signal known as frontal theta—a putative biomarker for cognitive control processes.

**Design, setting, and participants:**

Behavioral and EEG data were acquired from dental surgery trainees with 1 year (n=25) and 4 years of experience (n=20) while they performed low and high difficulty drilling tasks on a virtual reality surgical simulator. EEG power in the 4–7 Hz range in frontal electrodes (indexing frontal theta) was examined as a function of experience, task difficulty and error rate.

**Results:**

Frontal theta power was greater for novices relative to experts (p=0.001), but did not vary according to task difficulty (p=0.15) and there was no Experience × Difficulty interaction (p=0.87). Brain–behavior correlations revealed a significant negative relationship between frontal theta and error in the experienced group for the difficult task (r=−0.594, p=0.0058), but no such relationship emerged for novices.

**Conclusion:**

We find frontal theta power differentiates between surgical experiences but correlates only with error rates for experienced surgeons while performing difficult tasks. These results provide a novel perspective on the relationship between expertise and surgical performance.

**Key messages**

**What is already known about this subject?**

- Surgical training involves developing the ability to carry out complex sequences of action selection and execution processes. The majority of previous work has focused on the overt behavioral outputs of training, with little understanding of the underlying cognitive mechanisms that govern these processes.

**What are the new findings?**

- Using wireless, wearable electroencephalography (EEG) we examined changes in an oscillatory brain activity signal known as frontal theta as experienced and novice dental surgeons completed easy and difficult simulated surgical tasks. We show frontal theta can differentiate between surgical experiences, with higher frontal theta power for novices, but in a reversal of this global pattern, find signal power reduction with error commission in experts.

**How might these results affect future research or surgical practice?**

- These results point towards a more nuanced interpretation of the relationship between expertise and performance—one that may be modulated by cognitive control.
- In longer term, there is potential for EEG to inform training content to optimize learning and be used to monitor performance in the operating theater in real time.

## Introduction

Highly skilled surgeons have the ability to monitor and rapidly adapt to changes in the environment, appropriately tune into relevant information variables, select from a large repertoire of possible sensorimotor commands and execute with a smoothness that belies their many years of training.^1 2^ While the majority of research on surgical performance has examined the overt behavioral characteristics of such expertise (e.g., time to task completion^3 4^) and subjective measures of mental workload (largely examined through post hoc surveys^5^), there have been very few investigations into the underlying cognitive mechanisms that mediate the ability to carry out the complex sequences of action selection and execution required for surgical practice (see ref 6 for a review).

In cognitive neuroscience, the processes involved in goal-directed attention, outcome monitoring, executing motor commands and suppressing irrelevant motor responses are clustered under the label of ‘cognitive control’ (also referred to as ‘executive function’^7^). One putative neural correlate of cognitive control is a pattern of oscillatory brain activity known as ‘frontal theta’—a signal that can be observed on the scalp through electroencephalography (EEG) recordings and quantified by calculating signal power in the 4–7 Hz range.^8^

Frontal theta is considered to be critical in performance monitoring^9 10^ and core to error detection^10 11^—the key to triggering selection and prioritization of information processing^12 13^ and subsequent action.^14 15^ The recruitment of these ‘top down’ control processes is heightened in scenarios where automatic processes are insufficient for successful adaptation to the current environment,^7^ with the prefrontal cortex responsible for engaging a broad network of systems involved in goal-directed actions.^16–20^ To our knowledge, there has been no examination into the relationship between this neural signal and surgical performance to date.

Extant theories of skill acquisition often describe a shift from deliberate to automatic action selection and execution, with requisite reductions in the working memory requirements during the performance of a highly practiced action.^21^ A recent unifying framework for theories of cognition and action—known as the ‘Free Energy Principle’—proposes that the neocortex (involved in higher order functions, such as sensory perception and spatial reasoning) constantly makes inferences about the world and learns from experiences through the violation of its predictions.^22^ Viewed in this framework, frontal theta activity could serve as both a teaching signal for the system to learn that it needs to refine its prediction for the future and simultaneously, trigger the cognitive resources required to produce adaptive control.^23^ A more accurate world model would require fewer behavioral adjustments and thus, a reduction in the need to recruit cognitive control.

Predicated on this theory and evidence from neuroscience, we examined whether frontal theta activity could be used to distinguish between experienced and novice dental surgery trainees on a simulated drilling task carried out on a high-fidelity virtual reality simulator. We predicted that overall, novice participants would exhibit greater task-related theta activity, reflecting greater top-down engagement of cognitive control processes relative to their more experienced counterparts. Second, given that behavioral adaptation following prediction error is a hallmark of learning, we expected a relationship between performance errors and frontal theta activity.

## Materials and methods

### Participants

The data used for this study were obtained from undergraduate students of School of Dentistry, University of Leeds. The participants were assigned into two groups (as per their level of expertise). Forty-five participants were recruited with 25 first year dental students (the novice group: 16 female and 9 male, age=20.32±2.54 years) and 20 fourth year dental students (experienced group: 17 female and 3 male, age=23.7±0.58 years). Participants from both groups were recruited during the same period of time (over a 2-week period) through opportunity sampling and the experimenter was blinded to the group which participants belonged up until the point of testing. The sessions were booked through an online form where participants indicated the most suitable time during the hours the laboratory was available between 09:00 and 17:00. All participants were right-handed, provided informed consent and were fully debriefed.

### Experimental tasks

Participants completed the experiment on a haptic virtual reality dental simulator (Simodont; Moog FCS, Nieuw Vennep, Amsterdam, The Netherlands)). The simulator has previously been shown to have construct^24 25^ and predictive validity^26^ with real-world dental tasks. In this experiment, participants were asked to use the simulator to drill holes in two virtual shapes, with task difficulty operationalized as a function of the geometric complexity of the shape. The ‘Low Difficulty’ task involved drilling a simple straight shape, while the ‘High Difficulty’ task required participants to drill out a cross-shaped object.

**Figure 1:**
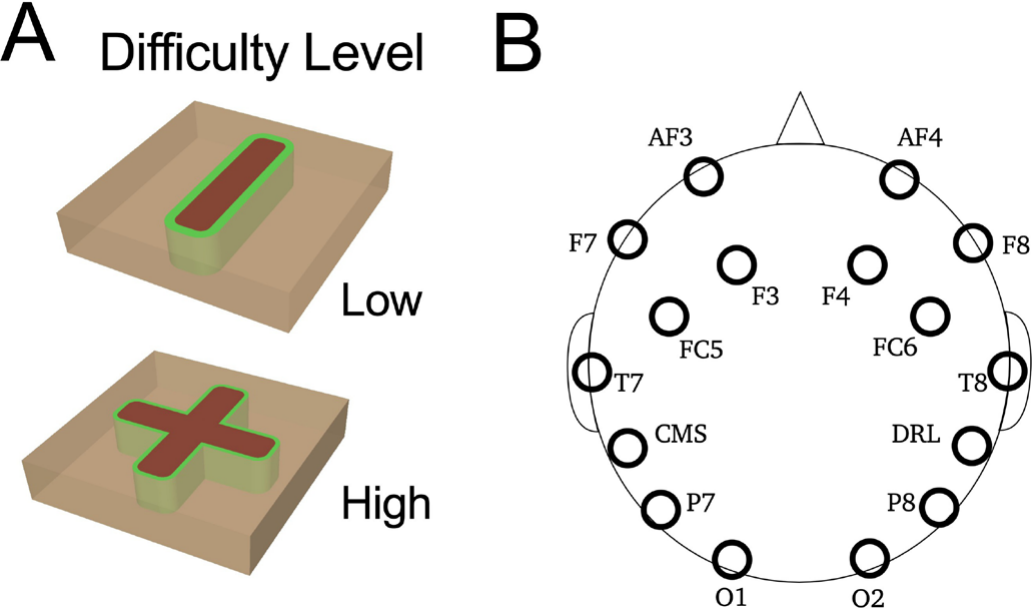
(A) Schematic drawing of the experimental tasks: the straight shape task is defined as a low level of difficulty while the cross shape presents a high level of difficulty. (B) Location of the electroencephalography (EEG) electrodes relative to head position. Analysis focused on channels F7, F8, AF3, AF4, F3, and F4.

Each shape comprised three regions: (1) a ‘Target’—participants were instructed that they must be removed; (2) a ‘Leeway’ area—which surrounded the target region on the sides and bottom (participants were instructed to avoid removing as much of this as possible); and (3) a ‘Container’ area on the sides and bottom surrounding the leeway zone and represented as a brown-colored region that participants were instructed that they must avoid during drilling (see figure 1). The goal for participants was drill/cut 99% of the target region while minimizing drilling in the leeway and avoiding the container regions as fast as they possibly could. To avoid potentially confounding order effects, we counterbalanced the presentation of the shapes across participants. Specifically, half the participants in each group performed the Low Difficulty task first followed by the High Difficulty task and the other half performed the High Difficulty task first followed by the Low Difficulty task.

### EEG acquisition device

EEG data were recorded using an Emotiv Epoc^+^ EEG wireless headset (Emotiv Systems, San Francisco, California). This system includes 14 active electrodes placed across the scalp according to the international 10-20 system (labeled AF3, AF4, F3, F4, F7, F8, FC5, FC6, P7, P8, T7, T8, O1 and O2) with two reference electrodes placed on mastoids bone behind the ear (common mode sense-left mastoid)/(driven right leg-right mastoid) ground (figure 1). The signal from each electrode was converted to digital form via a 16-bit analogy to digital converter, with a sampling frequency of 128 Hz.

### Procedure

All data collection was carried out in the Dental Simulation suite at the University of Leeds. The dental suite houses 30 simulators and is primarily designed for undergraduate and postgraduate dental students to practice. To provide a controlled environment for this study and to minimize the impact noise, each experiment took place in isolation, with the experimenter (AMB) testing one participant on one simulator at a time. The total duration of the study for each participant was approximately 15–20 min. Once participants were comfortably seated at the simulator, the EEG recording system was placed on the heads according to the manufacturer’s instructions. At this point (and prior to recording), participants received an introduction (lasting approximately 5–10 min) to the simulator from the experimenter and the tasks to be performed (figure 1). The EEG data were recorded continuously during the first dental task until the participants achieved the target performance level identified by the dental simulator. There was a 2 min break between the first and second tasks for all participants and the order in which participants completed the tasks was counterbalanced between participants.

### Data analyses

#### EEG data analysis

As the EEG signals observed on the scalp are inherently noisy, we undertook a number of preprocessing steps before statistical analysis. Raw EEG data were preprocessed for artifact removal and band decomposition using Brain Electrical Source Analysis (BESA; MEGIS Software, Gräfelfing, Germany). A linear finite impulse response filter was used to band pass the data between 1 and 20 Hz to remove the DC offset, line noise and other unwanted signals beyond the region of interest. Artifacts in the data were eliminated using an automatic artifact rejection routine implemented in BESA known as the ‘surrogate method’, which takes a multiple source approach to correcting eye artifacts and models brain activity using a fixed dipole method.^27^

Following this, we isolated theta band oscillations (4–7 Hz) from the channels in the frontal region of the scalp (F7, F8, AF3, AF4, F3, F4) and band power was computed every quarter of a second using Welch’s method, which estimates the power spectra based on a fast Fourier transform.^28 29^ It is important to note here that while we could also have extracted estimates for other frequency bands associated with sensorimotor planning and performance (eg, alpha,^30 31^ beta^32^), we had no strong a priori hypotheses for doing so in this particular study. Given the limited sample size available, coupled with the multidimensional nature of EEG signals—which allow researchers large analytical df,^33^ we opted against such analyses to avoid inflating the chances of type II error. Finally, our measure of frontal theta was computed by averaging activity across the frontocentral region. This improved the signal-to-noise ratio by minimizing the impact of any one single channel. One participant from the novice group had excessively noisy EEG data, with values more than three times the SD of the mean in the high and low difficult tasks, and was thus excluded from all analyses.

#### Statistical analysis

For behavior, we measured performance on the total error (quantified as percentages of drilling in the leeway regions). All measures were tested for normality to ensure the data met requirements for valid analysis of variance (ANOVA), by Q–Q plots and Shapiro-Wilk test. A 2×2 mixed ANOVA was conducted to compare performance across expertise (Novices vs Experts) × Task Difficulty (High vs Low) for each dependent variable. Correlation analyses were used to examine the relationship between frontal theta and the amount of error in the behavioral data for both the Low Difficulty and High Difficulty tasks for each group using a Pearson correlation. Comparisons of the magnitude of two correlations were performed where significant correlation was found using Hittner, May and Silver’s (2003)^34^ modification of Dunn and Clark’s (1969)^35^ approach using a back-transformed average Fisher’s Z procedure in the ‘cocor’ package for R.^36^ The statistical significance threshold was set at p<0.05 and we report generalized eta squared (*η*_G_^2^) as a measure of effect size and considered *η*_G_^2^=0.02 to be small, *η*_G_^2^=0.13 medium and *η*_G_^2^=0.26 to be a large effect size. To provide a measure of between-subject variability, we calculated coefficient of variation (CV) scores by dividing the SD of all scores from the mean of all scores and expressed as a percentage by multiplying this value by 100 for each condition: (σ/μ)*100. To capture within-subject variability between the Low Difficulty and High Difficulty tasks, we calculated an intrasubject CV, where for each participant, the SD of the two observations was divided by the mean of those observations and multiplied by 100. The mean average CV% value across participants was taken as a measure of within-subject variability of the group. All statistical analyses were performed using R V.3.5.2 (20 December 2018) and RStudio V.1.1.463.^37^

## Results

**Figure 2:**
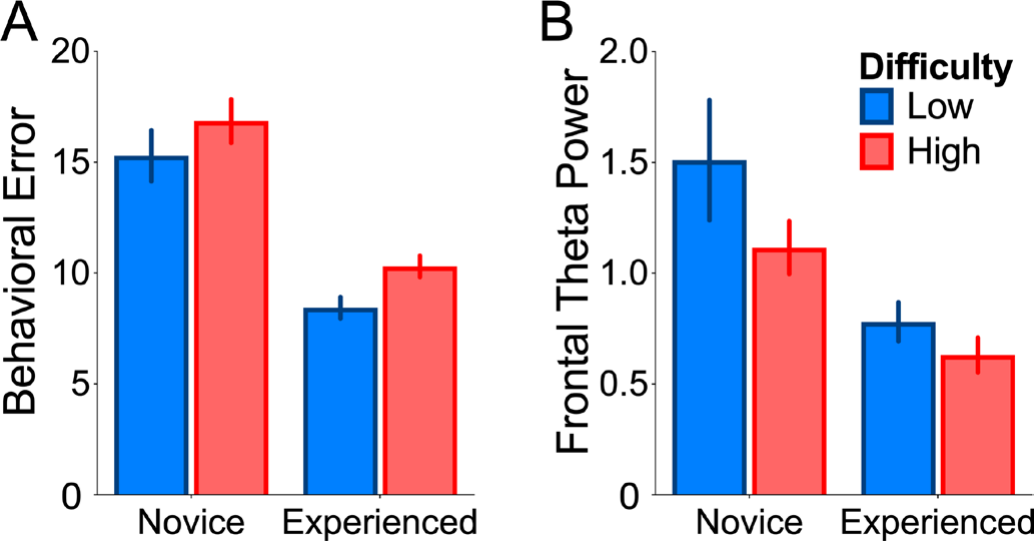
Group and task-related differences in behavior and neural activity. (A) Experienced participants made few errors relative to novice participants and participants made fewer errors in the Low Difficulty task relative to the High Difficulty task. (B) On average, the experienced group showed lower theta activity relative to novice participants. There was no reliable difference in theta activity as a function of task difficulty.

For behavioral performance (figure 2A), we found a significant effect of group (*F*(1,42)=41.18, p<0.001, *η*_G_^2^=0.41). As expected,^24^ error rates were higher for novice participants (M=16.51, 95% CI 14.99 to 18) relative to the experienced participants (M=9.68, 95% CI 8.16 to 11.2). There was also a significant effect of task difficulty (*F*(1,42)=5.3, p=0.03, *η*_G_^2^=0.04), with more errors occurring in the High Difficulty task (M=13.9, 95% CI 12.6 to 15.1) relative to the Low Difficulty variant (M=12.3, 95% CI 11 to 13.6). There was no interaction between task difficulty and group (*F*(1,42)=0.16, p=0.69, *η*_G_^2^=0.001). Novice participants in the Low Difficulty condition had the highest intersubject CV score (37.71%), which reduced to 28.60% in the High Difficulty condition. Experienced participants’ CV scores also reduced from Low Difficulty (25.90%) to High Difficulty (20.98%). For intrasubject CV we found marginally more variability in the novice group (19%) relative to the expert group (16.89%).

For our EEG measure of cognitive control (figure 2B), there was a significant main effect of group (*F*(1,42)=12.05, p=0.001, *η*_G_^2^=0.15), with frontal theta activity greater for novices (M=1.23, 95% CI 1.02 to 1.43) relative to experienced participants (M=0.72, 95% CI 0.52 to 0.93), indicating an increase in the recruitment of cognitive control. However, there was no difference across tasks (F(1,42)=2.11, p=0.15, *η*_G_^2^=0.02) and no interaction (*F*(1,42)=0.03, p=0.87, *η*_G_^2^<0.001). It is notable that for frontal theta power, we observed much larger degree of dispersion of scores across the group means compared with our behavioral error measure. The most variability was observed in the novice participants in the Low Difficulty condition (score 89.19%), which reduced to 52.24% in the High Difficulty condition. For experienced participants, the easy task had an intersubject CV score of 50.43%, which increased marginally in the High Difficulty condition (55.71%). The intrasubject CV was also larger for this measure relative to error, with a score of 32.68% for novices and 27.61% for experienced participants.

**Figure 3:**
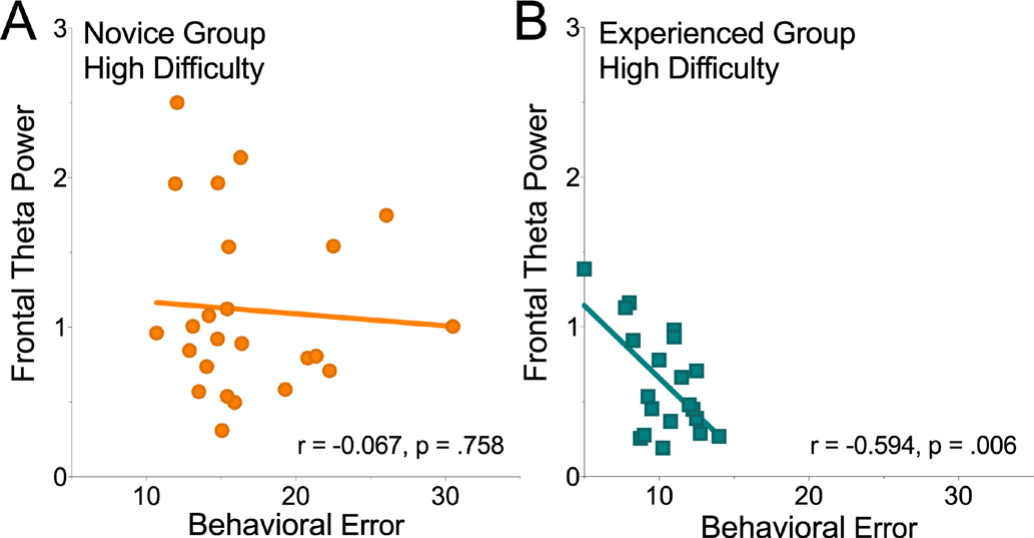
The relationship between behavioral performance and neural activity. Panel (A) shows no correlation between task error and frontal theta, but the experienced participants in the High Difficulty task exhibited a strong negative correlation indicating that better performance was linked to greater theta activity (B).

Finally, given the variability in our data, we explored whether the amount of frontal theta activity exhibited by participants could be correlated with the amount of behavioral error within each of our groups and across the two tasks. We found a significant negative correlation between frontal theta activity and behavioral error rates in the experienced group while completing the High Difficulty task (r=−0.594, n=20, p=0.0058). In other words, smaller behavioral errors were associated with greater theta activity while higher error rates were correlated with lower theta activity in this High Difficulty task for expert participants. We found no other statistically significant relationships (r’s<0.16; p’s>0.46). To examine whether this observed relationship between neural activity and task error for our experienced group in the High Difficulty task was significantly greater than the pattern found in the novice group (figure 3), we compared the magnitude of the two correlations and confirmed that the patterns were reliably different from one another (z=2.1779, p=0.0294).

## Discussion

Expert surgical performance is marked by seemingly effortless, flexible behavior^38^ that typically manifests in smoother movements, shorter operating times and fewer errors.^39 40^ This behavior is the product of a distributed network of neural circuitry that takes a complex sequence of action selection and planned motor execution plan and refines over time to ensure smooth and seemingly automatic performance.^41 42^ However, there have been very few investigations into the neural processes linking brain and behavior in the surgical domain,^43–51^ with technological constraints limiting the ability to probe the neural underpinnings of surgical performance.

We took advantage of recent advances in wireless EEG technology to understand these processes in more detail. We reasoned that a specific neural signal, referred to as frontal theta, a putative marker of cognitive control, would distinguish between experienced and novice dental students. We hypothesized that novice participants would require the recruitment of more cognitive resources to carry out the task relative to their more experienced counterparts and in line with this expectation, we found an increase in frontal theta activity for the novice.

We also found that the relationship between this signal and error correction was specific only to experts when performing difficult tasks. These results were not predicted a priori, and the result appears to indicate a reversal in the relationship between theta activity and performance and as such it is worth considering the nature of this relationship in detail. Here, in contrast to the global pattern in which theta power was greater for the novices relative to the experts, we found larger theta power for experts who made fewer errors. To understand the processes underlying this phenomenon, consider the example of a learner driver stepping into the driving seat for the first time. We can imagine that our student is keen to learn and thus extremely attentive to the stimuli visible on the road ahead. However, it is also clear that there is a limit to the performance levels that could reasonably be expected of our student—irrespective of the amount of attentional resources that might be recruited for the task. In this context, making the driving conditions more hazardous is unlikely to modulate the relationship between cognitive control and performance to any reasonable degree, given that performance levels are low and attentional allocation is already high. Now contrast this with a more experienced driver, fewer attentional resources are required relative to the learner to exercise a higher level of performance. But if we heighten the task demands, say through poor weather conditions, we can reason that those who make fewer errors are also likely to be the individuals with increased allocation of attentional resources. Note that for this analogy to apply to the present results, our experienced drivers should make enough errors for sufficiently worse performance, but not to the point of task failure. Indeed, on average, our experts’ behavioral performance far exceeded that of the novice participants.

These results also speak to a more general issue of the relationship between expertise and performance. Expert surgeons are often, as in this study, considered a homogenous group and their performance benchmarked against trainee groups.^52 53^ The present results indicate the existence of a more nuanced perspective on the relationship between experts and performance. While on average, their performance may be better than trainees (on metrics relating to time and error), the performance of any one individual is likely to be modulated by a number of factors. While the present data do not speak to causality, they do indicate a correlation between the amount of attentional allocation and performance in experienced participants and this may be a factor to consider in future comparisons—from experimental design (eg, motivation and distraction) to measurement and statistical analysis (examining heterogeneity within expert groups).

Some limitations of the present work are worth noting: while our sample size was comparable to the majority of previous research in this area, future research with larger sample sizes that have sufficient power to test the reliability of the brain—-behavior relationships identified here would be welcome. Reproducibility is the hallmark of science and we encourage replication tests of the results reported in this experiment. It is also important to note that our sample comprised dental surgery trainees who differed in 3 years of experience. Exploring these relationships across different levels of experiences and specialties will be important in testing the generalizability of these findings.

Given the increasingly lower costs of EEG technology and the ease in which these newer wearable systems can be incorporated within surgical simulation it is worth considering the potential benefits of doing so. While the recording of EEG in real-world settings is very much in its infancy, evidence is growing on the potential utility of this approach across a range of settings that could be instructive for surgical settings. For example, EEG signals are commonly used to trigger brain–computer interface robotic devices to support movement rehabilitation following stroke.^54^ Central to this approach is the need for individuals to be able self-regulate brainwave frequencies—a skill that is developed through neurofeedback training. This neurofeedback approach has also been used to optimize performance in neurologically healthy populations in a variety of tasks (see ref 55 for a review) with some work on developing surgical skills.^56^ Examining the long-term impact of such training for surgical skill development is key to advancing the implementation of these technologies.

With EEG providing a continuous measure of neural activity with temporal precision in the order of milliseconds, the signal could also be used to monitor performance and inform the provision of training content. Preliminary work has shown theta power can track changes in mental workload in military settings.^57^ With the majority of surgical incidents related to cognitive limitations,^58^ such an approach could potentially be used to self-monitor cognitive functioning in the operating theater. In the classroom, measuring frontal theta power may present an adjunct to existing measures of learning (which primarily focus on overt behavioral end-point performance) and allow trainers to modulate task difficulty to optimize skill acquisition within a training session.

To take advantages of the potential opportunities presented by low-cost wearable EEG, a more complete understanding of the neural processes underlying surgical skill acquisition and performance is necessary. The surgical education research community could benefit greatly from more frequently integrating this measure into the data collection process.

## Conclusion

In this study we show that frontal theta, a putative marker of cognitive control, can differentiate between surgical experiences. The data also indicate that frontal theta power scales with the degree of behavioral adjustment following error commission only for expert surgeons performing difficult tasks. These results point towards a more nuanced interpretation of the relationship between expertise and performance—one that may be modulated by cognitive control.
